# Research on Malodor Component Identification Based on Sensor Array

**DOI:** 10.3390/s25133857

**Published:** 2025-06-20

**Authors:** Jiaxing Xie, Wen Chen, Shiyun Chen, Peiwen Wu, Zhendong Lv, Jiatao Wu, Zihao Chen, Zonghong Li, Fan Luo, Xiaohong Liu

**Affiliations:** 1 College of Electronic Engineering (College of Artificial Intelligence), South China Agricultural University, Guangzhou 510642, China; xjx1998@scau.edu.cn (J.X.); 20223142031@stu.scau.edu.cn (W.C.); chenshiyu@stu.scau.edu.cn (S.C.); pven@stu.scau.edu.cn (P.W.); 13113118555@stu.scau.edu.cn (Z.L.); wjt_@stu.scau.edu.cn (J.W.); czh_@stu.scau.edu.cn (Z.C.); 13423567789@stu.scau.edu.cn (Z.L.); 20243172030@stu.scau.edu.cn (F.L.); 2 Engineering Research Center for Monitoring Agricultural information of Guangdong Province, Guangzhou 510642, China; 3 Shenzhen Yiyuntian Electronics Co., Ltd., Shenzhen 518126, China

**Keywords:** malodor, time series data, sensor array, short-term drift, continuous detection

## Abstract

With the rising demand for improved living standards and environmental protection, malodor pollution has emerged as a critical concern for both the public and regulatory authorities. Accurate prediction of malodor gas composition is essential for effective environmental monitoring and safety management. However, existing online malodor detection systems often suffer from short-term sensor drift, compromising their accuracy and long-term stability. To address these challenges, this study proposes an advanced electronic nose (e-nose) detection framework based on a time series data analysis. This study presents a novel approach utilizing a multi-channel sensor array for gas sampling, which establishes a robust mapping relationship between sensor response patterns and gas concentration distributions. To address the challenges of sensor drift and enhance system stability, we propose an innovative Encoder-Decoder architecture IED-CNN-LSTM incorporating external compensation mechanisms. Experimental results demonstrate that the proposed IED-CNN-LSTM model outperforms conventional methods significantly in both prediction accuracy and long-term stability. The framework achieves enhanced feature extraction from sensor time series data, enabling more precise and reliable detection of malodorous compounds. This research contributes an effective solution for real-time environmental monitoring applications while offering substantial improvements in both performance metrics and practical implementation for industrial and regulatory scenarios.

## 1. Introduction

Malodor refers to various smelly gases that can directly impact human health and mental state through the respiratory system [1]. With the rapid growth of the global economy, environmental pollution has become an increasingly urgent issue, and controlling malodor pollution has garnered widespread societal attention [2]. Currently, the scientific enforcement requirements for environmental monitoring are continually improving, placing higher demands on malodor detection. Simultaneously, controlling malodor requires accurate gas concentration data [3], which aids in identifying pollution sources [4], assessing pollution levels [5], analyzing real-time malodor diffusion [6], and formulating precise and effective control measures.

Traditional malodor detection methods can be broadly categorized into two types: artificial smell detection and component concentration analysis [7,8,9,10]. However, due to the limitations of these methods, it is difficult to meet the actual continuous detection application requirements. In contrast, gas sensor detection methods have the advantages of portability, high accuracy, and high cost-effectiveness and have been widely studied in the field of gas detection [11,12,13,14].

To improve the detection performance of gas sensors, many researchers have made significant progress in addressing nonlinear issues using machine learning methods [15,16]. To enhance the information content of gas sensor response curves, the intermittent sampling method is commonly used in data collection tests [17]. It has been found that gas sensor response processes exhibit clear temporal structure characteristics [18]. Consequently, numerous studies have explored time series signals of sensor responses using deep learning algorithms to achieve better detection performance. CNN is highly effective at capturing local spatial features in sensor sampling data, making the model’s input features more comprehensive and thus enhancing detection accuracy in practical tasks [19,20,21,22,23,24,25]. Since Recurrent Neural Networks (RNNs) can leverage the temporal nature of data and learn from temporal dependencies, studies have indicated that RNNs and their variants are well-suited for sequence modeling and particularly effective for processing predictive output tasks of sequence data [26]. Wang NN et al. [27] used LSTM networks to classify and identify gases from gas sensor array data, achieving better detection results than CNNs. Yu Cong et al. [28] combined gas data and environmental parameters to develop a feature-aware LSTM model for predicting pollutant gas concentrations effectively applicable to multi-component harmful gas detection. To further integrate the strengths of CNN and RNN, it has been found that combining the local feature extraction capability of CNN with the time series prediction ability of RNN can effectively extract temporal and spatial features from time series signals, facilitating the detection of complex gas structures [29,30].

Currently, gas sensors are still in the developmental stage. In practical applications, these sensors can be influenced by environmental interference and issues related to sensor stability, leading to unpredictable drift directions that are challenging to forecast [31,32,33]. In malodor detection, only a few studies have explored the application of deep learning methods in scenarios involving continuous changes in gas composition and concentration, while most studies rely on a single deep learning method [14,34,35,36]. The accuracy of real-time detection is crucial for information processing and subsequent applications [37,38], making the accuracy of short-term drift correction and online detection vital for practical application effectiveness. In common signal preprocessing methods, prior sensor information is required for drift correction [39,40]. Studies have shown that drift compensation algorithms based on prediction networks have practical value in real-world applications [41]. This method saves pre-drift information using a prediction network and calculates post-drift samples to achieve accurate detection results.

Building on the aforementioned research, this paper proposes an enhanced gas detection network model based on time series data for the detection of composite malodor gases. This method employs a sensor array for multi-channel sample complex malodor gases and constructs a model between the response fingerprint set and gas distribution results using preprocessing techniques such as feature filtering, feature selection, and sensor selection, achieving high-precision malodor measurement. In this study, eight types of malodor gases and their multi-component mixtures, following the common standard, are used as research objects [42], and an enhanced network model named IED-CNN-LSTM is constructed using the Encoder-Decoder architecture and an external compensation model. The Encoder-Decoder architecture serves as the primary model structure, with CNN and RNN structures utilized to extract the temporal and spatial features of the response signal. Additionally, the external compensation model processes sensor signal timing data and environmental information timing data using a Transformer network structure to enhance the extraction of deep signal information, thereby achieving precise compensation. The enhanced IED-CNN-LSTM network model leverages the advantages of time series prediction and parallel architecture, making it well-suited for continuous detection tasks. This study aims to optimize the component detection of malodor gases and provide an effective method for continuous detection of malodor gases.

## 2. Materials and Methods

### 2.1. Overview of Data and System Configuration

Sources of malodor in human production and daily life primarily include agriculture and livestock farming, industrial chemical production, and municipal facilities. This study analyzes and examines eight regulated malodor substances specified in the National Malodor Pollution Control Standard, as well as single gases and multi-component mixtures found in common life scenarios, to meet current malodor detection requirements [2].

To obtain the necessary data for the research, this study employs a self-developed electronic nose testing system comprising an air pump, gas washing and purification device, gas mass flow controller, electronic nose chamber, gas sensor array, signal conditioning circuit, data acquisition unit, and computer. The gas sensor array integrates 21 original channels of gas sensors (including MOS, EC, and PID principles), pressure sensors, and temperature/humidity sensors, as detailed in Table 1. The data acquisition unit consists of a host computer and multiple slave machines, allowing flexible addition or removal of sensor channels. In the hardware system, the MSP430F47187 chip serves as the main control module, and the sensor array in the gas chamber reacts with the target gas to generate electrical signals. The electrical signal is converted, amplified, and filtered by the signal conditioning circuit; then digitized by the AD converter; and finally transmitted to the main control chip via the UART serial interface. The main control chip communicates with the host computer via the UART serial interface to facilitate data transmission and storage.

### 2.2. Data Collection

**Figure 1:**
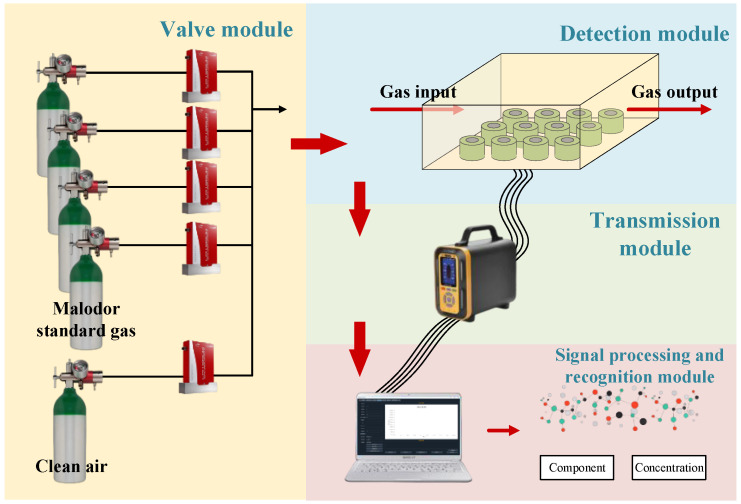
Schematic diagram of gas sensor array test system.

For acquiring standard concentration gas data, the developed electronic nose test system was utilized. The system’s device structure, shown in Figure 1, includes a gas distribution module, detection module, and signal processing and pattern recognition module. By modifying the gas distribution module structure, it can be configured for either data acquisition and testing equipment or the actual malodor detection system. The gas distribution system can mix various concentrations of target gas according to usage requirements to simulate real detection scenarios. In this study, the dynamic gas distribution method is employed for gas distribution steps [43], and the control of gas paths, flow rates, and gas component concentrations is achieved through host computer control.

In continuous malodor gas detection, considering sensor lifespan and the acquisition of response information, intermittent continuous detection is a more effective method [44]. However, in practical applications, the continuous sampling detection method is more widely used. This method involves an online detection system without gas path cleaning or preprocessing. As a non-intermittent continuous detection technique, it offers high real-time performance, a simplified detection system, and lower operational costs. This paper extends the discussion from intermittent to non-intermittent continuous detection to meet better application requirements.

Given the system’s simplicity and miniaturization requirements, clean air is used to flush the sensor and gas chamber. At the start of detection, the micro air pump is disconnected after a certain period, and clean air is then introduced to flush out any residual measured gas. In detection scenarios with higher real-time requirements, gas path cleaning pretreatment is not applicable, and non-intermittent continuous detection is used instead. Continuous passage of the target gas enables the sensor response, adapting to the sporadic and instantaneous nature of malodor gas in practical applications as well as the rapid changes in pollutant composition and concentration.

To ensure stable data acquisition, the sensor array is preheated for over 24 h before the response test to achieve a stable operating state. During the test, the composition and concentration of the gas mixture are automatically calculated and controlled by a dynamic gas distributor, maintaining a gas flow rate of 400 mL/min. The test steps for intermittent continuous detection are as follows: (1) clean the test chamber with clean air for 5–10 min and collect the sensor array response; (2) introduce the target gas into the sensor array gas chamber for 3 min to obtain the sensor array response; (3) clean the gas chamber again with clean air for 5–10 min to remove any residual target gas. The array’s response signal is recorded once per second, yielding 600 data points per cycle test. To simulate the actual detection process of the electronic nose, the above steps are repeated to achieve continuous malodor gas detection. Finally, several sample response datasets are obtained, forming Dataset I. The test steps for non-intermittent continuous detection are as follows: (1) pass clean air into the air chamber for 20 min and collect the sensor array response; (2) set the gas distribution system to random concentrations and ventilation times, and introduce the target gas into the sensor array gas chamber to obtain the sensor array response. The array’s response signal is recorded once per second, and data points reflecting the changes in malodor gas are continuously obtained throughout the test. Finally, the time series response data are obtained, forming Dataset II.

## 3. Methods

### 3.1. The Problem Description

The purpose of this paper is to achieve continuous detection of malodor gases in the environment and output the concentration information of eight regulated malodor substances. Given a fixed window for the continuous response data of the sensor, the training matrix *X* = {x_1_, x_2_, …, x_t_} ∈ R^n^ contains the sensor response values, temperature, humidity, and other variables, where *n* is the dimension of the relevant variables. We provided the concentrations of 8 regulated malodor substances during training; *y* = {y_1_, y_2_, …, y_8_}. Generally, we trained a nonlinear mapping function using the training matrix *X* and its associated target value *y* to obtain the predicted value *y*, formulated as follows:

(1) y=f(X)

Here, the nonlinear mapping function *f* is what we need to build and learn, and its accuracy is the primary focus and demand of the current research. For online application equipment, the environment is complex, with rapid changes in gas composition and concentration, and the sensor’s reaction time to the target gas is insufficient to obtain a complete response. Consequently, traditional data processing methods cannot effectively process the response signal. Moreover, regardless of whether intermittent or non-intermittent detection is used, the detection equipment may be affected by the previous working conditions, leading to short-term drift. Therefore, the constructed model must handle high-dimensional data and fully extract features based on the current and past states of the sensors to provide continuous and accurate detection methods. With the advent of the big data era and the rapid development of edge computing, these advancements provide a realistic basis for constructing complex models that can meet the needs for accurate malodor gas detection and build stable models for various gas detection methods.

### 3.2. Data Preprocessing

The electronic nose detection system often experiences “lag time,” which includes the delay in gas sensor response and gas path transmission. The sensor’s response and acquisition times are influenced by multiple factors, including chemisorption, physical adsorption, cumulative exposure, and the presence of target and interference gases [45]. In this study, the lag time was set as a hyperparameter [46], and the appropriate value was determined by analyzing the sensor response changes upon the target gas input, ensuring the sensor response matched the true gas concentration.

Additionally, gas sensors often encounter issues such as noise errors during detection and transmission. To maintain the authenticity of the original data and reduce undesirable noise and interference in the time series data, this study employed state estimation and smoothing filtering algorithms to process and analyze real-time data, providing accurate system state estimation and a smooth output. The specific preprocessing step involved sequential filtering using the Kalman filter [47] and the Savitzky-Golay filter [48]. First, the Kalman filter estimates the state and produces preliminary filtering results. Then, the preliminary filtered data are further processed and smoothed using the Savitzky-Golay filter to obtain the final pre-processed data. The purpose of this filtering scheme is to estimate the current system state using real-time information prior to the current time, thereby obtaining a more reliable state estimation. Smoothing reduces uncertainty due to sensor measurement errors or emergencies and enhances the tracking accuracy of the electronic nose detection system for actual state changes.

### 3.3. The Processing and Calculation of Gas Sample Data

For Dataset I, 3650 groups of response data were obtained, with each group containing changes in the cleaning process and the detection gas response from the last and current collections. Traditional detection methods construct models by extracting response curve characteristics from the current gas collection’s detection and cleaning processes [49]. This method leverages the response and recovery characteristics of gas sensors to comprehensively extract information from the target gas [50]. In this study, deep learning methods are used to automatically extract depth information features and historical change information from gas sensor response time series data, achieving high-precision multi-channel predictions. Test data are recorded once per second, and the response data from 3650 sample groups form a feature space of 3650 × 600 × channels, used as the input feature for the IED-CNN-LSTM model.

For Dataset II, 1205 continuously changing working conditions were generated, with 20 min of clean air passed at the start or end of each test to maintain the sensor array’s baseline level. Ultimately, 216,900 sets of time series response data were obtained. Test data were recorded once per second, and the dataset was split into 3-min intervals as input samples. This resulted in 7230 samples, forming a feature space of 7230 × 180 × channels used as the input feature for the IED-CNN-LSTM model.

The learning process of the IED-CNN-LSTM model consisted of two stages: training and testing. First, dynamic response data were collected from the gas sensor array, with 80% allocated to the training set and 20% to the test set. For the discontinuous detection data of Dataset I, the training set and test set were randomly divided into 2920 and 730 samples, respectively. For the non-discontinuous detection data of Dataset II, 80% of the data were allocated to the training set and 20% to the test set, resulting in 5784 and 1446 samples, respectively. The model learns potential patterns, relationships, and features from the training set.

Before training the model, data normalization can accelerate convergence, enhance the model’s generalization ability, and reduce the adverse effects of extreme values. Min-max normalization was used to standardize the dataset, as shown in the following equation:

(2) $$X_{normalized}=\frac{X-\min(X)}{\max(X)-\min(X)}$$

where *X_normalized_* represents the normalized value of the input feature *X*, and min(*X*) and max(*X*) represent the minimum and maximum values in the dataset, respectively.

During the training process, standardized samples from the training set were input into the IED-CNN-LSTM model to obtain the prediction output. The loss value between the predicted output and the actual value was calculated using the objective function, and the model parameters were continuously optimized through backpropagation to obtain the final model. The obtained model was then applied to the test set to verify its performance. The test set data also had to be standardized before inputting them into the model to generate the output results.

To verify the validity of the subsequent data and the model, it is necessary to extract features from the data. To this end, this study focused on feature extraction from the high-accuracy discontinuity detection Dataset I to complete subsequent model optimization. To extract comprehensive features, both time-domain and frequency-domain features of the response signal were extracted. Time-domain features included the response integral, differential, average differential, maximum, minimum, and average values [51]; frequency-domain features included the barycenter frequency, average frequency, and maximum frequency.

### 3.4. Feature Selection and Channel Selection

To efficiently detect malodor gases, optimize the system model size, and minimize the negative impact of redundant information from ineffective sensors on subsequent data analysis, channel selection was necessary. To select sensor channels, this study first identified the minimum number of features required for stable system detection through feature selection, ultimately determining the sensor channel input for the model.

For feature selection, this study employed an integrated feature selection method to comprehensively select the optimal combination, conducting a feature selection analysis using the feature extraction data from Dataset I. The filtering method (Pearson, MIC), embedding method (RF), and wrapper method (SVM-RFE) [52] were used, and the ranking of each feature selection method was obtained by calculating and analyzing the features and predicted output. The ranking set was comprehensively selected using the mean ensemble method to obtain the subset of key features. The ranking of features after feature selection can be expressed as follows:

(3) $$Rank_{FS_{n}}=\left|\begin{array}{c} {rank}_{{FS}_{n},{feature}_{1}}\\ \vdots \\ {rank}_{{FS}_{n},{feature}_{m}} \end{array}\right|$$

(4) $$\begin{array}{ll} Rank_{FS} & =\left|\begin{array}{ccc} Rank_{FS_{1}} & \ldots & Rank_{FS_{N}} \end{array}\right|\\ & =\left|\begin{array}{ccc} {rank}_{{FS}_{1},{feature}_{1}} & \ldots & {rank}_{{FS}_{N},{feature}_{1}}\\ \vdots & \ddots & \vdots \\ {rank}_{{FS}_{n},{feature}_{m}} & \ldots & {rank}_{{FS}_{N},{feature}_{m}} \end{array}\right| \end{array}$$

(5) $$Rank=\frac{1}{N}*\left|\begin{array}{c} \sum_{N}Rank_{feature_{1}}\\ \vdots \\ \sum_{N}Rank_{feature_{m}} \end{array}\right|$$

where *Rank_FSn_* represents the feature ranking obtained by the *i*-th feature selection method, ${rank}_{{FS}_{n},{feature}_{m}}$ represents the ranking of the *feature_m_*, and $Rank_{FS}$ represents the ranking of each feature under different feature selection methods. Finally, the rankings of each feature under different feature selection methods were summed and averaged to obtain the final feature ranking. Four feature selection methods were used in this study, where *N* = 4 and *m* = 9 × 21.

Finally, based on the feature ranking, the final ranking of each sensor was calculated, the sensor channel for the model was selected, and the input data structure of the model was determined according to the selected input channel, as follows:

(6) $$Sensor\_Rank=\frac{1}{m^{\prime }}\left|\begin{array}{c} \sum_{m^{\prime }}rank_{sensor_{1},feature}\\ \vdots \\ \sum_{m^{\prime }}rank_{sensor_{S},feature} \end{array}\right|$$

where *Sensor_Rank* is the ranking of sensor channels, *m*′ is the number of features extracted from each sensor, and *S* is the total number of sensors.

Based on the obtained *Sensor_Rank*, an ordered set was derived to select the channel response data that are effective for the model output. Finally, through the selection of sensor channels, the sensor response data of S1, S2, S3, S4, S5, S6, S7, S10, S11, S12, S13, S14, S15, S16, S17, and S19 and environmental information sensors S22 and S23 were selected.

### 3.5. The Structure of the System Model

**Figure 2:**
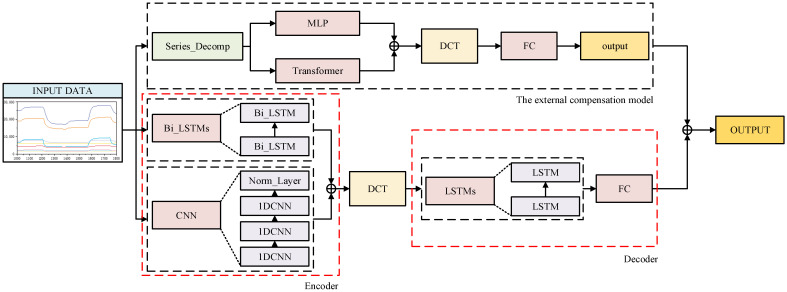
The diagram illustrates the structure of the IED-CNN-LSTM model.

Based on the suitability of current deep learning models, this study examines the data format, online detection methods, and detection requirements of gas sensors, constructing an IED-CNN-LSTM model that integrates CNN, RNN, Transformer, and Encoder-Decoder architectures. To adapt to the gas detection requirements under complex conditions, the model is illustrated in Figure 2.

The IED-CNN-LSTM model consists of a main structure and an external compensation model, with the main structure based on the Encoder-Decoder architecture. In the main structure, the flexibility and powerful modeling capabilities of the Encoder-Decoder architecture are leveraged to capture complex sequence features using a hybrid CNN and RNN model, ensuring the stability of the system network output. The external compensation model utilizes the Transformer’s capability to handle long sequences to optimize the output accuracy of the main structure. IED-CNN-LSTM leverages the strengths of each component to achieve a more stable structure and accurately predict the output of complex malodor gases from gas sensors.

The main structure encodes the input sensor channel time series data through the Encoder to extract deep features. The CNN block comprises three layers of one-dimensional CNN and one Norm_Layer, with non-linear connections established through the ReLU layer. The Bi_LSTM block consists of two Bi_LSTM layers. The CNN block and Bi_LSTM block are connected in parallel for data fusion. The dynamic response signal of the sensor serves as the input feature for the Encoder, and after encoding, the output is sent to the DCT-Attention [53] to compute the Decoder input. The Decoder consists of an LSTM block and multiple fully connected layers (FCs). The LSTM block comprises two LSTM layers. The input data undergo one-way processing, and the results are fed into the fully connected layer to perform model regression and obtain the concentration information of gas components. By fusing CNN and Bi_LSTM, the temporal and spatial characteristics of the sensor array response are captured simultaneously, yielding the initial model output.

In the external compensation model, the decomposition approach is used to break down the sensor time series response data into seasonal and trend components. Deep features are extracted from the gas sensor time series response data and the temperature, humidity, and pressure sensor time series response data, providing an accurate basis for output compensation. Following the basic structure of TDformer [54], the input data is decomposed, with trend data calculated using the MLP structure and seasonal data computed using the Transformer layer. The outputs are then fused in parallel. The fusion results are used for DCT-Attention calculations to enhance data feature extraction. The calculated results are flattened and input into the fully connected layer to obtain the concentration compensation value from the model. By compensating for the initial model output, the predicted concentration information of gas components is finally obtained.

Deep learning methods like RNN and CNN have significant advantages in handling high-dimensional data, with strong feature extraction capabilities, and can combine basic features layer by layer through a multi-layer network structure to form more advanced and effective information [55,56]. Compared to traditional machine learning models, they typically exhibit better performance [57]. Notably, the Encoder uses a parallel fusion of the CNN block and Bi_LSTM block, leveraging the bidirectional feature extraction of the Bi_LSTM model and the local feature extraction of the CNN to thoroughly learn sequence data features and ensure the stable output of the main structure.

### 3.6. Evaluation Criteria

Model evaluation metrics provide a clear representation of the correlation between predicted and actual values, allowing for a comprehensive assessment of model performance. To account for both error and fit, we selected three evaluation metrics: root mean square error (RMSE), coefficient of determination (R^2^), and correlation coefficient (CORR) to assess model accuracy. The specific descriptions are as follows:

(7) $$RMSE=\sqrt{\frac{1}{n}\sum_{i=1}^{n}{\left(\hat{y_{i}}-y_{i}\right)}^{2}}$$

(8) $$R^{2}=1-\frac{\sum_{i}{\left(\hat{y_{i}}-y_{i}\right)}^{2}}{\sum_{i}{\left(\bar{y_{i}}-y_{i}\right)}^{2}}$$

(9) $$CORR=\frac{\sum_{i}\left(y_{i}-\bar{y_{i}}\right)\left(\hat{y_{i}}-\bar{\hat{y_{i}}}\right)}{\sqrt{\sum_{i}{\left(y_{i}-\bar{y_{i}}\right)}^{2}\sum_{i}{\left(\hat{y_{i}}-\bar{\hat{y_{i}}}\right)}^{2}}}$$

where $y_{i}$ represents the actual value of fine particle concentration, $\hat{y_{i}}$ represents the predicted value, $\bar{y_{i}}$ represents the mean of actual values, $\bar{\hat{y_{i}}}$ represents the mean of predicted values, and *n* represents the number of predictions. RMSE measures the difference between predicted and true values. A smaller RMSE indicates better model performance. R^2^ measures the model’s ability to explain the variance of the dependent variable. R^2^ ranges from 0 to 1, with values closer to 1 indicating better model fit. CORR measures the correlation between observed and actual values. Higher CORR values indicate better model performance.

## 4. Results and Discussion

### 4.1. Descriptive Statistics

The data collected in this study are designed to simulate real-world collection scenarios. Clean air used in the experiments was sourced from an air compressor, ensuring no interference with the detection environment. Gas detection data from April to October were collected using an automatic acquisition method, including both daytime and nighttime data during the continuous acquisition process. The overall temperature range was 15 °C to 36 °C, and relative humidity ranged from 30% to 84%. A pressure sensor monitored traced pressure changes in the gas chamber during the detection process. In selecting malodor concentrations for gas distribution, both the current levels of malodor pollution emissions and actual experimental conditions were carefully considered. The specific concentration range is provided in Table 2. The aim is to expand the domain space of the collected data as much as possible to meet current detection needs. Studies have shown that the large spatial variability of the dataset enhances the model’s prediction and generalization abilities [58], indicating that the model developed in this study will perform well and better adapt to malodor detection equipment.

**Figure 3:**
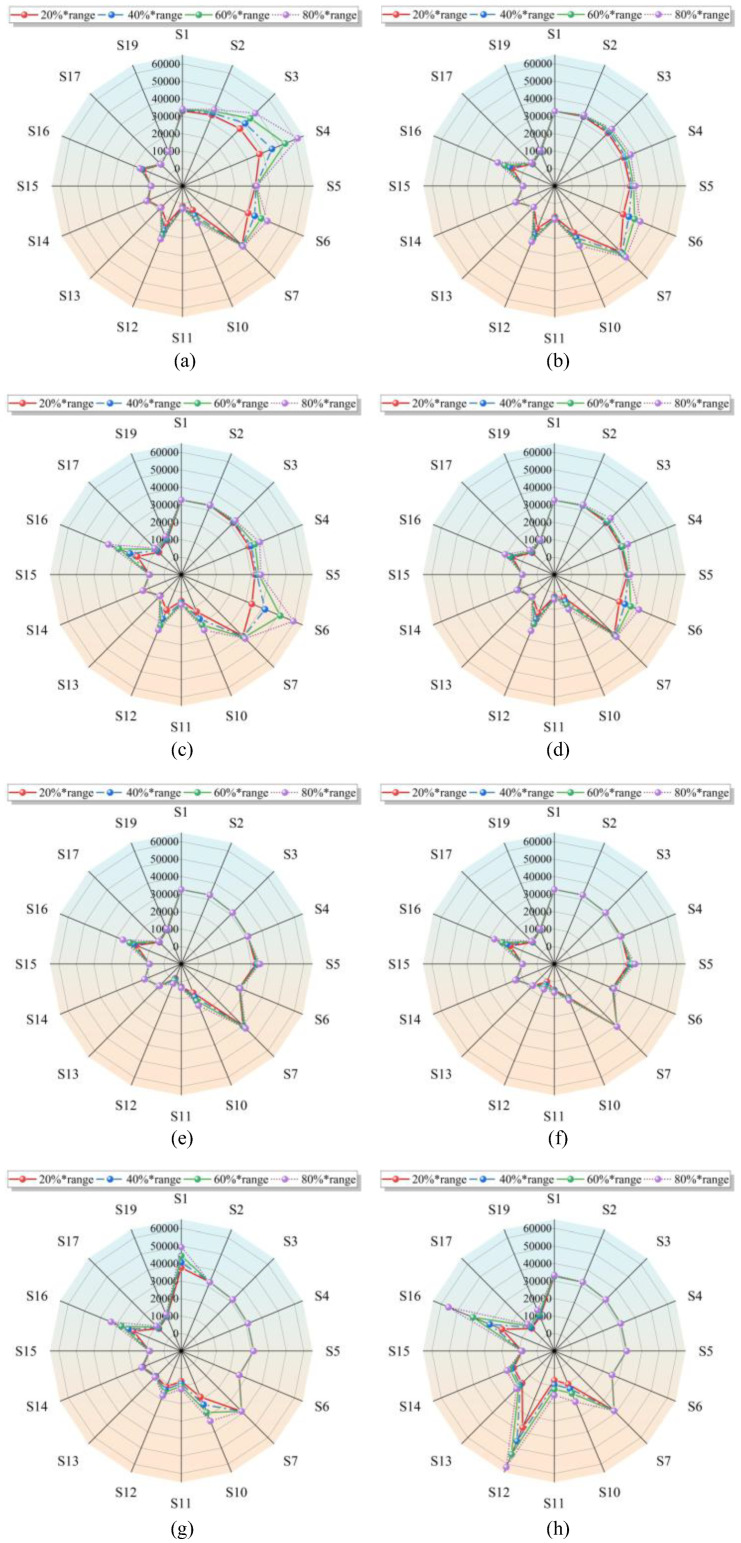
The radar map of malodor gases: (**a**) hydrogen sulfide gas information; (**b**) methyl sulfide gas information; (**c**) dimethyl disulfide gas information; (**d**) methyl mercaptan gas information; (**e**) carbon disulfide gas information; (**f**) styrene gas information; (**g**) ammonia gas information; (**h**) trimethylamine gas information.

To explore pattern recognition relationships, sensors selected for the channels were used as feature inputs for the model, covering eight target malodor gases and their concentration ranges. As shown in Figure 3, the response radar chart of the sensor array was exposed to eight controlled malodor gases at concentrations of 20%, 40%, 50%, and 80% of their respective ranges. The response values in the figure represent the stable voltage AD values recorded during the testing process. Most sensors exhibited distinct response patterns to different gases, and the response radar maps show significant differences and specificities. Comparing the sensor responses revealed that S4 is specific to hydrogen sulfide, S6 to dimethyl disulfide, S12 and S16 to trimethylamine, and other sensors also exhibit distinct differences for each malodor gas. Compared to other gases, the response patterns of (b), (d), (e), and (f) in the figure are similar but still exhibit differences. Achieving accurate detection from subtle feature differences is crucial for improving gas detection equipment. It is evident that individual sensor features form the basis of pattern recognition, and the feature sequence extracted by the sensor array is feasible for recognizing multiple types of malodor gases. This also provides a data foundation for constructing pattern recognition algorithms for complex multi-component gases.

**Figure 4:**
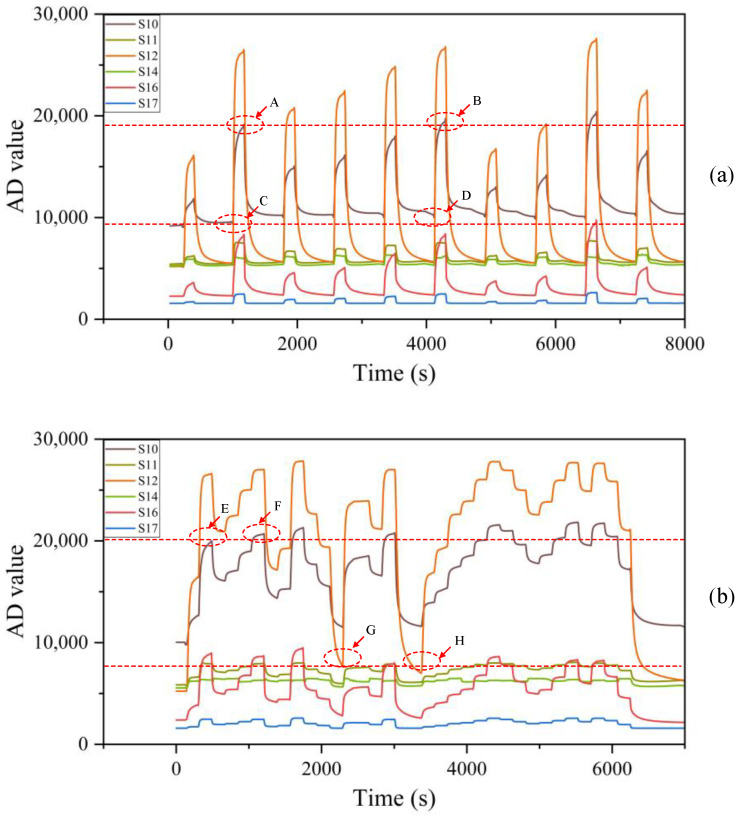
Sensor array response curve of methyl sulfide: (**a**) intermittent detection; (**b**) non-intermittent detection.

In this study, the collected data are transmitted from the lower computer to the upper computer for analysis. However, the detection performance and recovery characteristics of MOS, EC, and PID sensors are inconsistent. During continuous operation, these sensors can be influenced by preceding and subsequent detection processes, leading to significant errors in the current gas detection results and severely compromising detection accuracy. As illustrated in Figure 4a, positions A and B were affected by prior detection. Despite identical input concentrations, these positions exhibited different AD change values. Similarly, positions C and D were influenced by prior detection, which prevented the sensors from fully recovering. This resulted in error accumulation in subsequent detections and significant deviations in actual detection results. In Figure 4b, positions E and F demonstrate that sensor detection results can be affected by cumulative historical concentration errors during continuous detection. This led to markedly different AD changes at the same concentration. During the recovery process, comparing positions G and H revealed that concentration output results may be further affected if the sensor fails to promptly return to its optimal zero state during continuous detection. Regardless of the type of gas sensor, cumulative errors can occur during the detection process, highlighting the need for improved sensor design and calibration strategies to enhance detection accuracy and reliability.

### 4.2. Models Selection and Parameters Selection

In intermittent detection scenarios, sensor time series data can extract more comprehensive gas detection information. Traditional detection algorithms and deep learning algorithms both offer unique advantages in intermittent detection scenarios. To verify the effectiveness of the IED-CNN-LSTM model for concentration prediction, we utilized optimized traditional machine learning models, including K-Nearest Neighbor (KNN) [59], Support Vector Machine (SVM) [60], Random Forest (RF) [61], XGboost [16], and Multi-Layer Perceptron (MLP) [62]. These models, being supervised learning methods, have demonstrated superior performance in previous studies. To ensure the effectiveness of traditional methods, the high-performance Optuna framework was selected for hyperparameter optimization, thereby enhancing the performance of each model [63].

Based on the hyperparameter optimization methods and actual test results, the optimal parameters and hyperparameters for each model were as follows: KNN’s *k* value was set to 4, with neighbor sample weights determined by “distance”; SVM used a nonlinear RBF kernel function with a penalty coefficient of 50; RF had 700 base learners, a maximum decision tree depth of 9, and a random seed of 42; XGBoost had 850 weak learners, a learning rate of 0.025, a maximum model depth of 4, and a penalty coefficient of 0.026; and MLP consisted of two hidden layers with 300 and 150 neurons, respectively, used the Adam optimizer with a learning rate of 0.0004, and had a maximum of 100 iterations.

For the data collected in both current intermittent and non-intermittent detection scenarios, traditional data processing methods are no longer suitable for handling response signals. Therefore, the IED-CNN-LSTM model proposed in this paper can process the time series data collected by the sensors, manage the abnormal state of data drift during long-term detection, and provide more accurate prediction outputs. To verify the effectiveness of the improved IED-CNN-LSTM model in predicting the concentration of the two detection methods, the basic structure model of IED-CNN-LSTM was also used for comparison and validation. The models compared included CNN, LSTM, Bi_LSTM, CNN-Bi_LSTM, CNN-LSTM, ED-CNN-LSTM, and ED-CNN-DCT-LSTM. Notably, ED-CNN-DCT-LSTM constituted the main structural component of the system model, while ED-CNN-LSTM represented the main structural component without DCT-Attention. Additionally, to ensure the fairness of model validation, all common parameters across the models, such as hidden layer nodes, batch size, training epochs, and learning rate, were set to the same values to reflect the comprehensive performance of the IED-CNN-LSTM model as accurately as possible.

The algorithm was implemented using Python 3.8 in the PyCharm Community Edition 2022.1.4 integrated development environment on a Windows (×64) operating system with an Intel i5-11400H CPU, NVIDIA RTX 3050 GPU, and 4 GB RAM, utilizing the PyTorch 2.0.0 deep learning framework.

### 4.3. The Validation Results of the Model for Intermittent Detection

To evaluate the effectiveness of the IED-CNN-LSTM model for malodor detection in intermittent scenarios, we validated the preprocessed data from Dataset I and assessed the actual performance of each benchmark model. The preprocessed data were split into training and test sets at an 8:2 ratio. For traditional machine learning models, including the aforementioned channel feature selection, the data were further processed to extract artificial features. Experiments were conducted using the same hardware and datasets, and the model was evaluated on the same test set.

After examining the parameter indicators in Table 3, it is evident that despite the optimization of traditional machine learning models using Optuna, there remained a significant gap in performance metrics between discontinuity detection and deep learning models based on time series data. This suggests that traditional machine learning models face limitations in extracting dynamic information, leading to challenges in enhancing detection accuracy. In contrast, deep learning algorithms excel at extracting comprehensive feature information from time series data, allowing for greater improvements in model accuracy.

Further examination of the parameter indicators in Table 3 reveals that while single basic models (CNN, LSTM, Bi_LSTM) demonstrated good results and specific advantages, the hybrid model integrated the strengths of each model, achieving a higher upper limit of accuracy. Model testing revealed that the model incorporating the DCT-Attention mechanism achieved higher accuracy compared to the model without it. As shown in Table 3, compared to ED-CNN-LSTM, the RMSE index in the training set data decreased by 0.357, while R^2^ and CORR increased by 0.011 and 0.01, respectively. In the test set data, the RMSE index decreased by 0.351, and R^2^ and CORR increased by 0.008 and 0.009, respectively, indicating that the DCT-Attention mechanism significantly enhanced the model’s overall detection performance. Conventional time series models often overlooked the frequency information in the data. The DCT-Attention mechanism, however, focused on the temporal dependencies within time series data, highlighting the significance of feature extraction. The model with the DCT-Attention mechanism efficiently utilized frequency information, enhancing detection accuracy with minimal additional costs. This approach draws on the concept of FECAM [52] to enhance prediction accuracy and stability by integrating DCT with channel attention mechanisms to adaptively model frequency dependencies between channels.

Sensor-based malodor detection data often experience drift, reflecting changes in the actual conditions. Time series prediction models can effectively adapt to gas sensor detection. The primary structure model, built using gas sensor detection channel data, was combined with an external compensation model derived from environmental information sensor data to form a hybrid gas detection model, enhancing the model’s stability and accuracy. Table 3 demonstrates that, in the test set of Data I, the IED-CNN-LSTM model exhibited the best performance, with optimal RMSE, R^2^, and CORR indices after adding compensation. Compared to the ED-CNN-DCT-LSTM model without external compensation, the RMSE index in the training set data decreased by 0.522 ppm, while R^2^ and CORR increased by 0.01 and 0.007, respectively. In the test set data, the RMSE index decreased by 0.48 ppm, and R^2^ and CORR increased by 0.012 and 0.007, respectively. The IED-CNN-LSTM model effectively handled short-term drift during time series changes.

### 4.4. The Validation Results of the Model for Non-Intermittent Detection

Building on the accuracy advantages of intermittent detection, this study extends the methodology to non-intermittent detection. To validate the effectiveness of the ID-CNN-LSTM model for malodor gas detection in non-intermittent continuous scenarios, we tested on non-intermittent continuous detection data and evaluated the actual performance of each model using the preprocessed data from Dataset II. The time window affected model output. In this study, a 180-s sliding time window was found to perform better in terms of response data correlation and gas model stability. Based on this, the performance differences among various deep learning models were examined. The preprocessed data were split into training and test sets at an 8:2 ratio. Experiments were conducted using the same hardware and datasets, and the model was evaluated on the same test set.

According to the performance metrics in Table 4, the method based on intermittent detection can be effectively extended to non-intermittent detection, enabling continuous malodor detection using a gas sensor array. The single basic models (CNN, LSTM, Bi-LSTM) in the table demonstrate effectiveness, with high RMSE, R^2^, and CORR indices, indicating certain advantages. However, the hybrid model integrated the advantages of each individual model, achieving a higher upper limit of accuracy and offering greater competitiveness in high-precision continuous detection. Further examination of the parameter indicators in Table 4 reveals that the ED-CNN-DCT-LSTM model with the DCT-Attention mechanism achieved higher accuracy compared to the ED-CNN-LSTM model without it. Specifically, the RMSE index in the training set data decreased by 0.192 ppm, while R^2^ and CORR increased by 0.003 and 0.007, respectively. In the test set, the RMSE index decreased by 0.105 ppm, and R^2^ and CORR increased by 0.012 and 0.008, respectively.

In a continuously changing environment, the drift characteristics of sensor-based malodor detection data are more challenging to predict. Consequently, by extending the intermittent detection method, the primary structure model, built using gas sensor detection channel data, was combined with an external compensation model derived from environmental information sensor data, forming a hybrid gas detection model that continued to yield good results. Table 4 shows that the IED-CNN-LSTM model performed best in the test set of Data II. After adding compensation, the RMSE, R^2^, and CORR indicators were 0.486 ppm, 0.987, and 0.993 in the training set data, respectively, and 0.51 ppm, 0.984, and 0.99 in the test set data. Observing the IED-CNN-LSTM model and its comparison models, it is clear that the IED-CNN-LSTM model performed the most prominently. Adding the DCT-Attention mechanism, hybrid model structure, and external compensation structure progressively enhanced performance, further confirming the effectiveness of these mechanisms. This also demonstrates that the method based on discontinuity detection can be effectively extended to non-discontinuity detection. By integrating a time series model with an external compensation structure, the system can adapt to both intermittent and non-intermittent detection methods in current gas sensor malodor detection, meeting various detection requirements and real-time detection needs.

**Figure 5:**
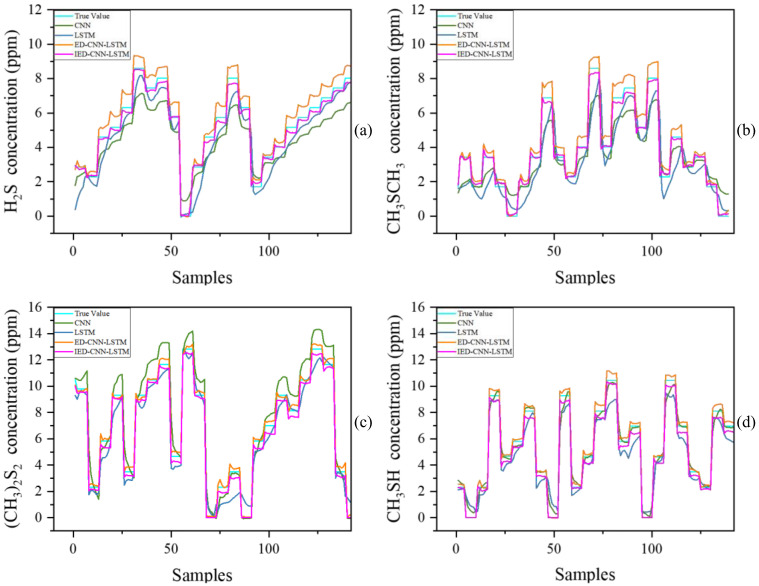
The concentration curves of malodor gases: (**a**) hydrogen sulfide gas testing set; (**b**) methyl sulfide gas testing set; (**c**) dimethyl disulfide gas testing set; (**d**) methyl mercaptan gas testing set.

In the actual continuous detection scenario, the concentration diagram of real values and predicted values is depicted in Figure 5, illustrating the concentration curves of the test set data for four sulfur-containing organic compound odor gases. As shown in the figure, the prediction accuracy of single deep learning models, such as CNN and LSTM, was significantly lower than that of hybrid deep learning models, particularly the ED-CNN-LSTM and IED-CNN-LSTM models. Throughout the detection process, the ED-CNN-LSTM and IED-CNN-LSTM models consistently achieved higher prediction accuracy. Notably, the IED-CNN-LSTM model exhibited superior accuracy and stability compared to other models, especially during periods of large input concentration changes. Generally, prediction accuracy is high when the gas concentration is relatively stable. However, it tends to decrease when the gas concentration changes rapidly. The results demonstrate that the IED-CNN-LSTM model, which is the focus of this study, achieved excellent performance regardless of whether the gas concentration was stable or undergoing rapid changes. This further validates the effectiveness and stability of the external compensation model and the hybrid model employed in this research.

### 4.5. The Discourse on Gas Sensor Detection

This study provides valuable insights into optimizing gas sensor devices for continuous malodor gas detection. It examines improvements in detection methods, data structure, and model innovation to meet current detection requirements. Both intermittent and continuous detection methods are practical and necessary; they balance the lifespan of the sensor with the need for real-time detection, offering effective solutions for malodor gas monitoring and catering to diverse current needs. Consequently, enhancing detection accuracy remains a critical research direction.

To improve detection accuracy, this study first preprocessed the sensor-collected data, followed by selecting sensor channels to simplify the characteristics of gas data. A hybrid Convolutional Neural Network (CNN) and time series model was then designed to analyze gas sensor process data. This led to the construction of a more stable mixed gas detection model and the proposal of an IED-CNN-LSTM model tailored for malodor gas detection. The IED-CNN-LSTM model demonstrated significant performance improvements over traditional machine learning models in both intermittent and continuous malodor detection datasets, showcasing its robust detection capabilities. The superior performance of our model is attributed to a hybrid optimization strategy that considers the stability and diversity of data, the effectiveness of data information, the process characteristics of gas sensors, the mining of sequential data information, and the efficacy of model integration. Based on these considerations, a hybrid model has been developed, achieving notable improvements in accuracy and complexity. This enhances the robustness, applicability, and precision of the model in complex detection scenarios.

Furthermore, we acknowledge the challenge of detecting drastic changes in malodor gas concentrations. According to the principles of Short-Term Exposure Limit (STEL) and Time-Weighted Average (TWA) [64], maintaining stable detection is crucial for current and future digital prevention and control efforts. This study identifies an effective sensor combination for extracting gas information from traditional detection methods and extends the continuous detection method by designing a deep learning model tailored to sensor time series response data. The proposed model adeptly captures the dynamic patterns of gas changes, thereby playing a pivotal role in reducing errors and enhancing detection accuracy.

## 5. Conclusions

This study focused on malodor gas detection, employing a conventional intermittent detection method to select optimal features and channels from a sensor array. This process effectively removes redundant data and enhances the performance of the gas sensor array. Building on this foundation, we utilized the time series response data from the gas sensor array to predict malodor gas concentrations, with the goal of providing high-precision model support for continuous detection systems. To accommodate both intermittent and continuous detection methods, we extended traditional intermittent detection techniques to continuous detection using an Encoder-Decoder architecture augmented with an external compensation model. By leveraging the strengths of Convolutional Neural Networks (CNNs) and time series prediction models, we constructed a model specifically tailored to gas sensor response data. This approach significantly improved the system’s overall prediction and recognition accuracy, enabling precise prediction of malodor gas identity and concentration in malodorous environments. The key conclusions of this study are as follows:

(1) Input data processing stabilized the system model. State estimation, data filtering, and lag processing were applied to sensor-collected data. Integrated feature selection based on intermittent detection data simplified gas data features. Deep learning models were utilized to extract time series data features, constructing a malodor system model based on time series data.
(2) To enhance the accuracy of continuous malodor detection equipment, we proposed the IED-CNN-LSTM model. Constructed using an Encoder-Decoder architecture and an external compensation model, this system integrates the benefits of CNNs and time series models, utilizing the DCT-Attention mechanism to improve detection accuracy. Two types of malodor gas data—intermittent and continuous—were collected to form datasets I and II. Tested on an NVIDIA RTX 3050, the IED-CNN-LSTM model achieved an RMSE of 0.236 ppm, R^2^ of 0.965, and CORR of 0.965 for intermittent detection (Dataset I) and an RMSE of 0.51 ppm, R^2^ of 0.984, and CORR of 0.99 for continuous detection (Dataset II). These results signify a successful transition from high-precision intermittent detection to continuous detection, improving the accuracy of non-intermittent continuous detection and enabling high-precision detection of multi-component mixed malodor gases.
(3) The implementation of a compensation model validated the feasibility and effectiveness of long-term continuous malodor gas identification and accurate concentration prediction. Optimized by the compensation model, the model enhanced time series data detection, improving gas detection model performance and offering practical algorithmic support for high-performance gas detection schemes. The extension from intermittent to continuous detection methods not only improved non-intermittent detection accuracy but also provided a viable solution for future malodor application research.

Future steps involve integrating this method into complex malodor gas detection equipment for practical application verification.

## Figures and Tables

**Table 1:** Information on the selected sensors.

ID	Sensor Type	Detection Principle	Manufacturer
S1	4NH_3_-100	EC	Honeywell (Morristown, NJ, USA)
S2	4H_2_S-100	EC	Honeywell (Morristown, NJ, USA)
S3	4CH_3_SH-10	EC	Honeywell (Morristown, NJ, USA)
S4	4C_2_H_6_S-10	EC	Honeywell (Morristown, NJ, USA)
S5	4CS_2_-10	EC	Honeywell (Morristown, NJ, USA)
S6	4C_2_H_6_S_2_-10	EC	Honeywell (Morristown, NJ, USA)
S7	CS_2_/MF-100	EC	Membrapor (Wallisellen, Switzerland)
S8	4C_8_H_8_-10	EC	Honeywell (Morristown, NJ, USA)
S9	4SO_2_-20	EC	Honeywell (Morristown, NJ, USA)
S10	TGS813	MOS	Figaro (Osaka, Japan)
S11	TGS2600	MOS	Figaro (Osaka, Japan)
S12	TGS2602	MOS	Figaro (Osaka, Japan)
S13	TGS2603	MOS	Figaro (Osaka, Japan)
S14	TGS2620	MOS	Figaro (Osaka, Japan)
S15	PID-A1	PID	Alphasense (Essex, Britain)
S16	PID-AH	PID	Alphasense (Essex, Britain)
S17	PID-TECH-200	PID	Baseline (Mesa, AZ, USA)
S18	PID-TECH-2000	PID	Baseline (Mesa, AZ, USA)
S19	RainbowPID-200	PID	AU (Boulder, CO, USA)
S20	RainbowPID-10000	PID	AU (Boulder, CO, USA)
S21	4R-NDIR	NDIR	Wuhan Zhituo (Wuhan, China)
S22	MS5803	RC	Intersema (Bevaix, Switzerland)
S23	EEH210	RC	E + E Elektronik (Engenwitzdorf, Austria)

**Table 2:** The information regarding the concentration range of collected malodor gases data.

Name	Range (ppm)
Hydrogen sulfide	0–14
Methyl sulfide	0–14
Dimethyl disulfide	0–14
Methyl mercaptan	0–14
Carbon disulfide	0–14
Styrene	0–14
Ammonia	0–20
Trimethylamine	0–20

**Table 3:** Evaluation results for intermittent malodor gas detection.

Data	Model	Training Set			Testing Set		
		RMSE	R^2^	CORR	RMSE	R^2^	CORR
Feature data	KNN	5.019	0.937	0.949	5.328	0.926	0.940
	SVM	7.020	0.912	0.921	7.863	0.904	0.917
	RF	5.655	0.934	0.930	5.736	0.926	0.927
	XGBoost	5.346	0.937	0.944	5.664	0.928	0.938
	MLP	3.672	0.949	0.952	3.942	0.936	0.946
Time series data	CNN	3.396	0.944	0.958	3.186	0.947	0.956
	LSTM	2.067	0.958	0.964	2.106	0.949	0.961
	Bi_LSTM	2.028	0.959	0.964	2.034	0.950	0.965
	CNN-LSTM	1.944	0.959	0.960	1.884	0.954	0.961
	CNN-Bi_LSTM	1.899	0.956	0.971	1.794	0.957	0.968
	ED-CNN-LSTM	1.605	0.966	0.974	1.563	0.967	0.973
	ED-CNN-DCT-LSTM	1.248	0.977	0.984	1.212	0.975	0.982
	IED-CNN-LSTM	0.726	0.987	0.991	0.732	0.987	0.989

**Table 4:** Evaluation results of deep learning models for non-intermittent malodor gas detection.

Data	Model	Training Set			Testing Set		
		RMSE	R^2^	CORR	RMSE	R^2^	CORR
Time series data	CNN	4.026	0.866	0.942	3.798	0.831	0.936
	LSTM	1.989	0.904	0.956	2.235	0.888	0.947
	Bi_LSTM	1.800	0.917	0.960	2.022	0.906	0.952
	CNN-LSTM	1.614	0.935	0.968	1.758	0.927	0.964
	CNN-Bi_LSTM	1.755	0.941	0.965	1.839	0.934	0.965
	ED-CNN-LSTM	0.822	0.972	0.974	0.792	0.968	0.971
	ED-CNN-DCT-LSTM	0.630	0.975	0.987	0.687	0.972	0.982
	IED-CNN-LSTM	0.486	0.987	0.993	0.510	0.984	0.990

## Data Availability

The data presented in this study are available upon request from the corresponding author.
